# Irisin Rescues Blood-Brain Barrier Permeability following Traumatic Brain Injury and Contributes to the Neuroprotection of Exercise in Traumatic Brain Injury

**DOI:** 10.1155/2021/1118981

**Published:** 2021-10-16

**Authors:** Peipei Guo, Zhao Jin, Jin Wang, Aming Sang, Huisheng Wu

**Affiliations:** ^1^Department of Anesthesiology, The First Affiliated Hospital of Anhui Medical University, Hefei City, Anhui Province 230022, China; ^2^Department of Anesthesiology, Zhongnan Hospital of Wuhan University, Wuhan City, Hubei Province 430071, China

## Abstract

Traumatic brain injury (TBI) has a high incidence, mortality, and morbidity all over the world. One important reason for its poor clinical prognosis is brain edema caused by blood-brain barrier (BBB) dysfunction after TBI. The mechanism may be related to the disorder of mitochondrial morphology and function of neurons in damaged brain tissue, the decrease of uncoupling protein 2 (UCP2) activity, and the increase of inflammatory reaction and oxidative stress. In this study, we aimed to investigate the effects of exogenous irisin on BBB dysfunction after TBI and its role in the neuroprotective effects of endurance exercise (EE) in mice. The concentrations of irisin in cerebrospinal fluid (CSF) and plasma of patients with mild to severe TBI were measured by ELISA. Then, male C57BL/6J mice and UCP2 knockout mice with C57BL/6J background were used to establish the TBI model. The BBB structure and permeability were examined by transmission electron microscopy and Evans blue extravasation, respectively. The protein expressions of irisin, occludin, claudin-5, zonula occludens-1 (ZO-1), nuclear factor E2-related factor 2(Nrf2), quinine oxidoreductase (NQO-1), hemeoxygenase-1 (HO-1), cytochrome C (Cyt-C), cytochrome C oxidase (COX IV), BCL2-associated X protein (Bax), cleaved caspase-3, and UCP2 were detected by western blot. The production of reactive oxygen species (ROS) was evaluated by the dihydroethidium (DHE) staining. The levels of inflammatory factors were detected by ELISA. In this study, we found that the CSF irisin levels were positively correlated with the severity of disease in patients with TBI and both EE and exogenous irisin could reduce BBB damage in a mouse model of TBI. In addition, we used UCP2^−/−^ mice and further found that irisin could improve the dysfunction of BBB after TBI by promoting the expression of UCP2 on the mitochondrial membrane of neurons, reducing the damage of mitochondrial structure and function, thus alleviating the inflammatory response and oxidative stress. In conclusion, the results of this study suggested that irisin might alleviate brain edema after TBI by promoting the expression of UCP2 on the mitochondrial membrane of neurons and contribute to the neuroprotection of EE against TBI.

## 1. Introduction

The huge advances in society, economy, transportation, and infrastructure have produced an increasingly convenient life, while resulted in people's higher probability of developing trauma [1, 2]. Accordingly, the incidence of traumatic brain injury (TBI) has been annually rising worldwide and remains at a continuingly high level. TBI, a common emergent and critical illness in clinic, bears an extremely high risk for disability and mortality and has an inadequately satisfying clinical prognosis. One of the main causes for a series of adverse outcomes is brain edema which is not only a consequence of TBI but also a major factor for further aggravating TBI damage [3].

TBI can destroy tight junction (TJ) proteins, the main structure of the blood-brain barrier (BBB), and cause the apoptosis of cerebrovascular endothelial cells. This allows substances that could not have access to the brain tissue to quickly permeate into it in large quantities, so that fluids accumulate in the extracellular space, resulting in vasogenic brain edema [4, 5]. The mechanism may be that reactive oxygen species (ROS) increase after TBI, which leads to neuroinflammatory response and oxidative stress response [6]. Meanwhile, the mitochondrial membrane potential (MMP) is a significant factor accounting for the increase of ROS levels, so MMP, to some extent, is positively correlated with ROS [7]. Recently, there is increasing evidence that uncoupling protein 2 (UCP2) can dramatically reduce the production of ROS by reducing MMP [8]. The “uncoupling survival” hypothesis related to UCP2 has been further confirmed in the traumatic brain injury model [9].

It is well-established that endurance exercise (EE) can effectively exert neuroprotective effects [10–13] but the underlying mechanism has remained to be elucidated. Exercised skeletal muscle can secrete PGC-1*α*, and downstream factors regulated by this protein can be sheared and modified to form a hormone called irisin. Irisin has been a focus of recent studies in the fields of metabolism and the nervous system [12, 14]. Our previous research has demonstrated that irisin worked effectively on alleviating cerebral ischemia-reperfusion injury [15]. Thus in this study, we tested whether irisin could reduce brain edema after TBI and explored its specific mechanisms. In addition, we also observed whether the neuroprotective effects of EE were related to irisin.

## 2. Materials and Methods

### 2.1. Clinical Study on Patients with TBI

A total of 16 adult patients with mild to severe TBI (GCS 9-15 points) were recruited from the Department of Neurosurgery at Zhongnan Hospital of Wuhan University. They were all qualified for the strict criteria for inclusion and exclusion. Samples of cerebrospinal fluid (CSF) and plasma from TBI patients were collected. The concentrations of irisin in CSF and plasma were measured by ELISA. The severity of TBI in patients at different stages was evaluated by the Glasgow coma scale (GCS), which mainly evaluated the degree of coma and trauma in terms of eye-opening response, verbal response, and limb movement. The total score is the sum of those three scores, and the highest is 15, indicating clear consciousness. A lower score is an indicator for more severe brain injury, closely related to the degree of cerebral edema [16].

All clinical procedures were performed following the protocol approved by the Ethics Committee of Zhongnan Hospital of Wuhan University, and the informed consent of all participants and legal guardians was obtained (registration number: ChiCTR1900025962, ethics batch number: 2018008).

### 2.2. Animals

Three-month-old adult male C57BL/6J mice and UCP2 knockout mice with C57BL/6J background, weighing 19-23 g, were all purchased from Saiye Biotechnology Company. The UCP2 knockout mice were the global knockout model. Before the experiment, mice were placed in the same environment for more than 3 days where the temperature was 20-25°C and the light/dark cycle was 12 h. They were allowed to take in food and water freely. All experimental operations were approved by the Animal Care and Use Committee of Wuhan University, in line with the guidelines of the National Institutes of Health Laboratory Animal Care and Use Guidelines.

### 2.3. Endurance Exercise

Motor treadmill (electric treadmill) exercise was adopted in this study to better simulate human exercise patterns. The mice exercised 5 days a week (Monday, Tuesday, Thursday, Friday, and Saturday). The protocol of exercise for the first week was as follows: 6 m/min × 30 min × 2 d, 8 m/min × 30 min × 2 d, 10 m/min × 30 min × 1 d. The protocol of daily exercise for the following 6 weeks was as follows: 6 m/min × 10 min + 8 m/min × 15 min + 10 m/min × 15 min + 8 m/min × 10 min + 6 m/min × 10 min. Daily exercise time of mice was restricted to 15 : 00-17 : 00 to prevent the interference of the circadian rhythm and the rhythm of the time point.

### 2.4. TBI Model

The controlled cortical impact (CCI) method was employed and slightly adjusted in our study to set up the TBI model, which is following the existing literature [17, 18]. Briefly, after 8 h preoperative fasting but free access to water drinking, mice were anesthetized with an intraperitoneal injection of 1.5% pentobarbital sodium 50 mg/kg. Then, the mice's skull skin was disinfected, and the tops of the right skulls were cut to reveal the junction of the sagittal suture and the coronal suture. Next, the periosteum was scraped, and a bone window with a diameter of about 4 mm was drilled at a distance of 2 mm from the sagittal suture and the coronal suture. The mice were then fixed on the base of the eCCI electronic craniocerebral injury instrument (RWD Life Science Co., Ltd.), receiving 120 ms' mild to moderate TBI impact with parameters set at 4 m/s speed and 2 mm depth. Meanwhile, in the Sham operation group, only the bone window was established and no operation was performed. Then, the bone fragments and the skin were sutured. Finally, the mice were reared in separate cages under ventilation and natural light.

### 2.5. Experimental Groups

Mice were randomly divided into six groups: (1) Sham group, a control group that subjected to sham operation; (2) TBI group, a TBI group that underwent mild to moderate TBI impact with parameters set at 4 m/s speed and 2 mm depth; (3) irisin-treated group (Ir), and Ir group that was injected with 10 *μ*g/kg irisin intravenously immediately after TBI impact; (4) EE group, an EE group that underwent 5 weeks of physical exercise before TBI impact; (5) EE/IgG-treated group (EE+IgG), an EE+IgG group that underwent 5 weeks of physical exercise before TBI impact and then injected with nonimmune control IgG intravenously; and (6) EE/NA-treated group (EE+NA), an EE+NA (neutralizing antibody) group that underwent 5 weeks of physical exercise before TBI impact and then injected with neutralizing antibody against irisin intravenously (20 *μ*g per mouse, Phoenix Pharmaceuticals).

### 2.6. Analysis of Mice's Weight and Survival Rate

When the TBI models of mice in the four groups (Sham group, TBI group, EE group, and Ir group) were successfully constructed, all mice were normally raised in an open and ventilated environment. The deaths of them were all spontaneous, and no euthanasia was performed. Death was defined as the following: the mouse had cardiac arrest, the collapse of the left atrium, and simple convulsions. A researcher who was unclear about the specific grouping observed the survival of the mice once a day. He timely kept records of deaths when observing the weight, the modified neurological severity score (mNSS), brain water content, and other indicators of mice at the corresponding time points.

### 2.7. Modified Neurological Severity Score

The modified neurological severity score (mNSS), commonly used in neurobehavioral evaluation after TBI, mainly detects mice's exercise ability, balance ability, alertness, and the like. Its score range is 0-18 points, and the score is in positive proportion to the severity of neurological damage. The test, carried out by a researcher who was unclear about the specific grouping, was on the day before TBI and 1, 3, 7, and 14 days after TBI. All mice in each group were tested for their neurological function, and they had been properly trained before modeling to adapt to this test.

### 2.8. Determination of Brain Water Content and Evans Blue (EB) Content

The brain water content was determined by the dry and wet weight method. The mice were decapitated, and the brains were collected 24 h after TBI. The brain was immediately weighed as wet weight and then was placed in a 110°C oven for 24 h and weighed for the dry weight. Brain water content (%) equals (wet weight − dry weight)/wet weight × 100%.

To determine the EB content, EB physiological saline solution was injected through the femoral vein 1 h before the mice were sacrificed. The injection was indicated successful when the eyeball conjunctiva and limbs all showed blue, and then, cardiac perfusion was performed after 1 h of circulation. After deep anesthesia, the left ventricles of the mice were perfused with normal saline to the right atrial appendage till clear liquids flowed out. The mice were decapitated, and the brain was collected, made into frozen sections, and immediately placed under an inverted fluorescence microscope with blue excitation light to observe the leakage of EB. A fluorescence spectrophotometer was used to measure the content of EB in brain tissue.

### 2.9. Immunohistochemistry

The paraffin sections (5 *μ*m thick) of mouse brain tissue were roasted overnight at 37°C and deparaffinized and incubated with 3% hydrogen peroxide for 15 min. After the thermal repair of the tissue antigens by microwave, the slices were cooled for 30 min at room temperature and incubated with rabbit anti-irisin antibodies (1 : 500; Phoenix Pharmaceuticals). Then, the protein expression of irisin was observed after biotin-labeled secondary antibodies, goat anti-rabbit IgG (1 : 100; KPL) incubation. Ten fields of each slice were observed under a light microscope (×400) by a blind pathologist. The integrated optical density (IOD) was measured by an Image-Pro Plus analysis system. The protein expression intensity was positively correlated with IOD.

### 2.10. DHE Staining

A frozen section of the brain tissue isolated from mice in different groups was incubated with dihydroethidium (DHE) which was diluted with PBS (10^−5^ M) at 37°C for 40 min and fixed with paraformaldehyde for 10 min. Then, DAPI staining solution was used to stain the nucleus for 10 min. Finally, the images were observed using a fluorescence microscope at an excitation wavelength of 490 nm and an emission wavelength of 590 nm, under which the blue-stained part was the nucleus, and the green fluorescence reflected the ROS content. The exposure time used for image acquisition of all sections was 30 ms. The DHE fluorescence intensity was quantified by ImageJ.

### 2.11. Western Blot

Briefly, the total proteins from the damaged brain tissue or blood were extracted by centrifugation at 4°C for 10 minutes. Then, the proteins were separated by SDS-polyacrylamide gel electrophoresis onto the nitrocellulose membrane and buffered and blocked with skimmed milk powder at room temperature for 1 h. The membranes were incubated with the rabbit anti-irisin antibody (1 : 1000; Abcam), the rabbit anti-occludin antibody (1 : 1000; CST), the rabbit anti-claudin-5 antibody (1 : 1000; Abcam), the rabbit anti-zonula occludens-1 (ZO-1) antibody (1 : 1000; CST), the rabbit anti-nuclear factor E2-related factor 2 (Nrf2) antibody (1 : 1000; CST), the mouse anti-quinine oxidoreductase (NQO-1) antibody (1 : 1000; CST), the rabbit anti-hemeoxygenase-1 (HO-1) antibody (1 : 1000; CST), the rabbit anti-cytochrome C (Cyt-C) antibody (1 : 1000; CST), the rabbit anti-cytochrome C oxidase (COX IV) antibody (1 : 1000; CST), the rabbit anti-BCL2-associated X protein (Bax) antibody (1 : 1000; CST), the rabbit anti-cleaved caspase-3 antibody (1 : 1000; Abcam), and the rabbit anti-uncoupling protein 2 (UCP2) antibody (1 : 1000; CST) at 4°C overnight. After being washed with TBST, the membranes were incubated with the secondary anti-rabbit or anti-mouse IgGs (1 : 50000; KPL) at room temperature for 30 min. Optical density analysis for quantification was performed on the ImageJ software (NIH).

### 2.12. ELISA

The levels of irisin in the plasma and CSF of patients with TBI were measured at 2 and 7 days after injury following the manufacturer's instructions of the ELISA kit (Boster Co., Ltd, Wuhan). At different time points, the levels of irisin, malondialdehyde (MDA), superoxide dismutase (SOD), and glutathione peroxidase (GSH-PX) in the brain tissue of mice and the levels of inflammatory factors (TNF-*α*, IL-1*β*, IL-4, and IL-10) in plasma and brain tissue of mice were detected by ELISA (Boster Co., Ltd., Wuhan).

### 2.13. Transmission Electron Microscope

24 h after TBI, the mice were sacrificed under deep anesthesia. The brain tissue was quickly removed from the ice dish, and the damaged brain tissue was selected. The tissue was then fixed with 2% paraformaldehyde and 2.5% glutaraldehyde at 4°C for 2 h, rinsed 3 times with sodium arsenate buffer (pH 7.2) for about 10 min, fixed with 1% osmium acid (OsO4) at 4°C for 2 h, and rinsed again with double distilled water 3 times for about 10 min. After that, gradient alcohol dehydration was followed by isoamyl acetate replacement. Finally, the structure of endothelial cells, tight junctions, and neuronal mitochondria reflecting the BBB was observed under the transmission electron microscope (JEM-1011, JEOL).

### 2.14. Brain Microvascular Extraction, Mitochondrial Extraction, and MMP Determination

The mice were decapitated under deep anesthesia, and the injured part was removed and placed in a petri dish filled with cold phosphate buffer saline (PBS). The cortex was then extracted, cut into 1 mm size, homogenized, filtered through a 41 *μ*m pore size nylon filter, and washed 3 times with PBS. Then, the capillaries on the filter were collected and centrifuged before 15% dextran was added. The ingredients were combined thoroughly and suspended to 20% dextran, and another centrifuge was done to save cerebral capillary sediment at the bottom.

Capillaries were suspended and mixed with 2 ml 0.1% collagenase/dispase (containing 20 U/mL DNase I) and digested in a 37°C water bath for 1 h, centrifuged to remove the supernatant, and added 2 ml of culture medium to form a suspension. Then, they were spread on a 12 mL 50% Percoll solution that formed a continuous gradient after centrifugation. The purified microvascular segment was the white-and-yellow-colored layer above the red blood cell layer near the bottom. The segment was then extracted, rinsed twice with culture medium, and centrifuged. The supernatant was discarded, and the remainder was stored in liquid nitrogen for western blot.

The brain tissue around the lesion was extracted, and the mitochondria in it were separated using a mitochondrial extraction kit (NBP2-29448, NOVUSBIO). The protein concentration of each sample was adjusted to 1 g/L by using mitochondrial preservation solution, and the relative fluorescence unit (RFU) was obtained after detecting the mitochondrial membrane potential (MMP) *ΔΨ*m with the help of a fluorescence microplate reader, according to the instructions of the kit (GENMED, USA).

### 2.15. Statistical Analysis

All data were presented as mean ± SEM. Statistical analysis was carried out using SPSS 20.0 (SPSS Inc., San Rafael, CA, USA) and GraphPad Prism 5.0 (San Diego, CA, USA). Repeated measures analysis of variance (ANOVA) was used for multiple comparisons and analysis among groups, which was followed by Dunnett's post hoc test. The significance of correlations was evaluated by determining Spearman's rank correlation coefficients, and the difference of estimate of cumulative survival (Kaplan–Meier) among groups was evaluated with the log-rank test. Statistical significance was based on unpaired two-tailed Student's *t*-tests, with *P* < 0.05 indicating statistical significance.

## 3. Results

### 3.1. CSF Irisin Levels Were Increased and Positively Correlated with the Recovery of Disease in Patients with TBI

**Figure 1:**
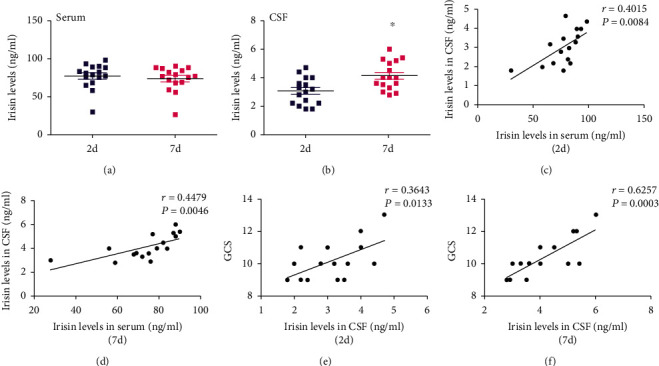
CSF irisin levels were increased and positively correlated with the recovery in patients with TBI. (a) The serum level of irisin was derived from patients who suffered from TBI for 2 days or 7 days. (b) The CSF level of irisin was derived from patients who suffered from TBI for 2 days or 7 days. (c) Correlations between CSF irisin levels and serum irisin levels in patients who suffered from TBI for 2 days. (d) Correlations between CSF irisin levels and serum irisin levels in patients who suffered from TBI for 7 days. (e) Correlations between GCS and CSF irisin levels in patients who suffered from TBI for 2 days. (f) Correlations between GCS and CSF irisin levels in patients who suffered from TBI for 7 days. *n* = 16. The significance of correlations was evaluated by determining Spearman's rank correlation coefficients. ^∗^*P* < 0.05 vs. TBI for 2 days.

As shown in Figure 1, the plasma irisin levels of TBI patients in the recovery period (7 d after trauma) were slightly lower than that in the early posttraumatic period (2 d after trauma), while the CSF irisin concentrations were significantly increased (*P* < 0.05, Figures 1(a) and 1(b)). In the early posttraumatic period and recovery period, the plasma irisin levels of TBI patients were positively correlated with the CSF irisin concentrations (*P* < 0.05, Figures 1(c) and 1(d)). All these results indicated that the CSF irisin concentrations of patients with TBI were related to their recovery.

Glasgow coma scale (GCS) is one of the most commonly used clinical indicators to assess the degree of trauma in TBI patients. In this study, it was found that the CSF irisin concentrations in TBI patients were positively correlated with GCS score (*P* < 0.05, Figures 1(e) and 1(f)) in the early posttraumatic and recovery period, suggesting that the change of CSF irisin levels may be one of the significant cause for affecting the prognosis of TBI patients.

### 3.2. Endurance Exercise Can Increase the Level of Irisin in the Brain Tissue of Mice

**Figure 2:**
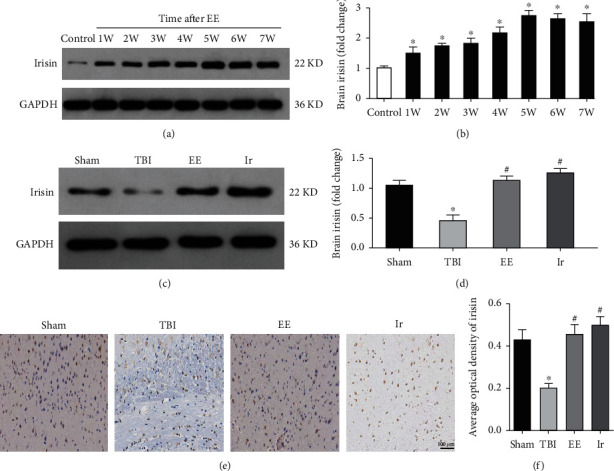
Endurance exercise could increase the level of irisin in the brain tissue of TBI mice. (a, b) The expression of brain irisin derived from mice underwent EE at a different time without TBI. (c, d) The expression of brain irisin derived from mice in different groups. (e, f) Immunostaining showed that irisin was detected in the brain tissue of mice in different groups. Data are expressed as the mean ± SEM. Scale bars: 100 *μ*m. *n* = 6. Significance was determined by one-way ANOVA and Dunnett's post hoc analysis. ^∗^*P* < 0.05 versus Sham group or control; ^#^*P* < 0.05 versus TBI group.

Western blot was used in our study to observe the level of irisin in the brain tissue of mice with or without TBI after they exercised for a different duration. As shown in Figures 2(a) and 2(b), the brain irisin levels increased continuously during 1-5 w of exercise (*P* < 0.05) and attained the peak at 5 w, with its value being almost 3 times that of nonexercise mice (control group). However, no significant change was found after another 2 w continuing exercise, basically maintaining the peak level and slightly decreasing. This indicated that the irisin level produced by mice in physiological state after 5 w exercise reached the peak, and any more exercise brought no significant increase, so 5 w was the duration of exercise common to all the experiments concerning exercise in our study. In addition, brain irisin levels in mice suffering from TBI were significantly lower than those in the Sham operation group, but after 5 w exercise, such levels were significantly higher than those of mice in the TBI group alone (*P* < 0.05, Figures 2(c)–2(f)). The above results suggested that exercise could increase the secretion of irisin in brain tissue.

### 3.3. Neuroprotection of Endurance Exercise Can Be Replicated by Application of Exogenous Irisin

It has been reported that endurance exercise exerts a protective effect on TBI, cerebral ischemia-reperfusion injury, and Alzheimer's disease [10–13], and irisin, as an important peptide in the body, can be secreted by skeletal muscle and brain tissue after endurance exercise [19]. This leads our study to observe whether endurance exercise and exogenous irisin have the same neuroprotective effect on TBI.

To test this hypothesis, we first observed the expression of irisin in the brain tissue of mice after exogenous irisin treatment by western blot and immunohistochemistry. As shown in Figures 2(c)–2(f), the brain levels of irisin significantly increased in the Ir groups compared with the TBI group (*P* < 0.05), which indicated that exogenous administration of irisin could promote it to enter the brain tissue.

**Figure 3:**
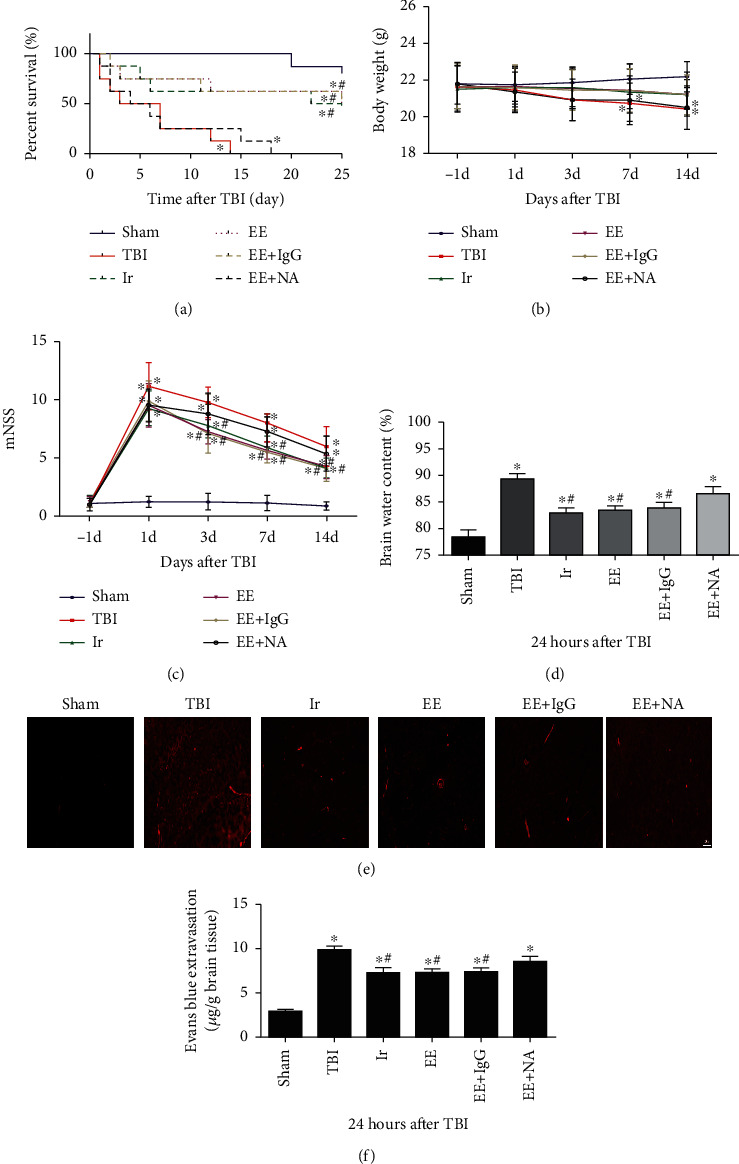
Endurance exercise and exogenous irisin have similar protective effects on mice with TBI. (a) The survival rate of mice in different groups. (b) The body weight of mice in different groups. (c) mNSS of mice in different groups. (d) The brain water content of mice in different groups. (e, f) The leakage of EB in brain tissue was observed by a fluorescence microscope in different groups. Data are expressed as the mean ± SEM. Scale bars: 20 *μ*m. *n* = 5. Significance was determined by one-way ANOVA and Dunnett's post hoc analysis. ^∗^*P* < 0.05 versus Sham group; ^#^*P* < 0.05 versus TBI group.

The TBI model used in this study results in severe damage to mice and thus high mortality, so it is of great significance to observe the survival rate of mice in different groups. As demonstrated in Figure 3(a), the survival rate of mice in the TBI group, EE group, and Ir group was significantly lower than that of the Sham group (*P* < 0.05), while the survival rate of mice in the EE group and Ir group was significantly higher than that of the TBI group (*P* < 0.05). There was no difference between the EE group and the Ir group (*P* > 0.05).

Animal weight is also an objective indicator reflecting the survival of mice in different groups. As illustrated in Figure 3(b), the weight of animals in the TBI group was significantly lower than that in the Sham group at 7 d and 14 d after TBI (*P* < 0.05), while at other time points, there was no difference in weight among different groups (*P* > 0.05).

The modified neurological severity score (mNSS) is often used to assess changes in behaviors after TBI. The higher score represents the more severe behavioral impairment. As illustrated in Figure 3(c), on the day before TBI, there was no significant difference in mNSS results among different groups (*P* > 0.05); at day 1, day 3, day 7, and day 14 after TBI, the mNSSs of the other 3 groups were higher than those of the Sham group (*P* < 0.05), while the mNSSs of the EE and Ir groups were lower than those of the TBI group at 3, 7, and 14 days after TBI (*P* < 0.05). Compared to the EE group, the mNSSs of the EE+NA group were higher at 3, 7, and 14 days after TBI (*P* < 0.05), while there was no difference between the mNSSs of the EE group and the EE+IgG group.

The peak period of brain edema after TBI is generally 24 h after trauma, and brain water content (BWC) and EB content are effective indicators to reflect the degree of brain edema. The results of Figures 3(d)–3(f) showed that the BWC and EB content in the brain tissue increased significantly after TBI (*P* < 0.05), while the BWC and EB content in the brain tissue of the EE group and Ir group decreased slightly, compared with those in the TBI group (*P* < 0.05). All these results suggested that both endurance exercise and exogenous Irisin could, to a certain extent, reduce neurological damage after TBI, and they exerted similar effects.

### 3.4. Exogenous Irisin Reduced the Damage of Ultrastructure and Upregulated the TJs after TBI

TJs form as an essential barrier when multiprotein complexes extend to the endothelial cell space to form a side-to-cell diffusion. The destruction of its integrity is greatly responsible for increased BBB permeability and brain edema. Hence, in this study, we observed the changes after TBI in the ultrastructure of microvasculature and some key proteins occludin, claudin-5, and ZO-1 that constitute TJ.

**Figure 4:**
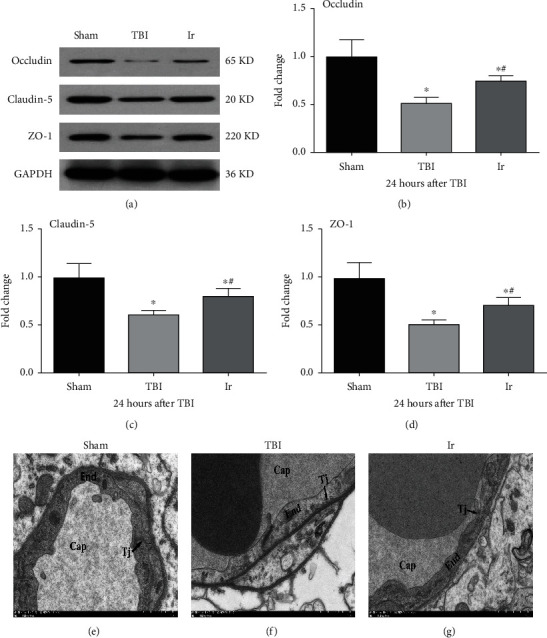
Effects of exogenous irisin on BBB integrity at 24 h after TBI in mice. (a–d) The expressions of occludin, ZO-1, and claudin-5 in brain tissue at 24 h after TBI using western blotting analysis. (e–g) Electron microscopic examination of microvasculature in the brain tissue at 24 h after TBI. Cap: capillary; TJ: tight junction; End: endothelial cell. Data are expressed as the mean ± SEM. Scale bars: 1.0 *μ*m. *n* = 6. Significance was determined by one-way ANOVA and Dunnett's post hoc analysis. ^∗^*P* < 0.05 versus Sham group; ^#^*P* < 0.05 versus TBI group.

As shown in Figures 4(a)–4(d), the expressions of occludin, claudin-5, and ZO-1 were significantly reduced after 24 h of TBI, compared with the Sham group (*P* < 0.05). With the treatment of exogenous irisin, the expressions of occludin, claudin-5, and ZO-1 were significantly upregulated than those in the TBI group (*P* < 0.05). These results suggested that exogenous irisin could promote the expressions of TJ-related proteins.

As shown in Figures 4(e)–4(g), the endothelial cells of the microvasculature had normal morphology without deformation and swelling, and the TJs of the plasma membrane of adjacent endothelial cells were intact, and no gaps were seen in the Sham group. In comparison, in the TBI group, capillary endothelial cells swelled and thickened significantly, and there were evident gaps in TJs between endothelial cells. Meanwhile, endothelial cells in the Ir group were slightly flat and had a slighter swelling than in the TBI group, and there was a certain gap in TJs between endothelial cells.

### 3.5. Exogenous Irisin Inhibited Inflammation and Oxidative Stress after TBI

TBI could cause a series of neuroinflammatory reactions, mitochondrial dysfunction, and oxidative stress, which was further followed by secondary destruction of the BBB, and a vicious circle of further aggravated inflammation. Therefore, we, respectively, observed the changes in the levels of proinflammatory and anti-inflammatory cytokines and the indicators of oxidative stress in the brain tissue and plasma after TBI.

**Figure 5:**
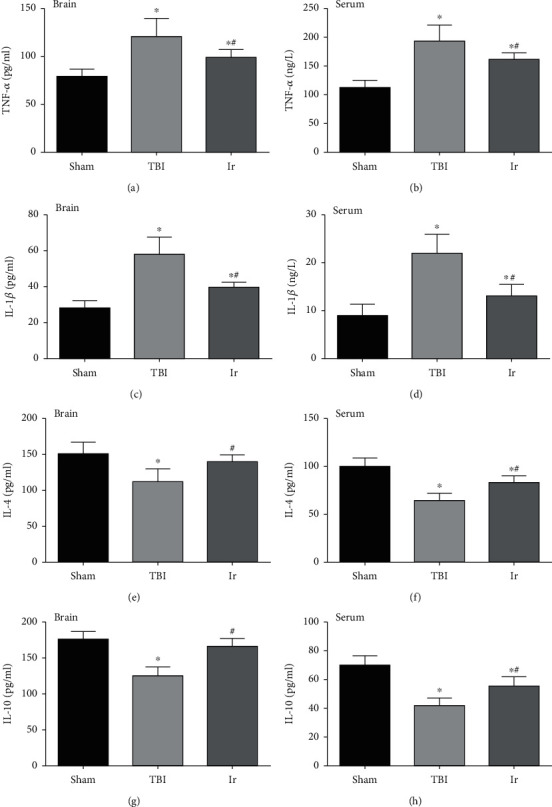
Effects of exogenous irisin on the inflammatory response at 24 h after TBI in mice. (a, c, e, g) Content of TNF-*α*, IL-1*β*, IL-4, and IL-10 in brain tissue at 24 h after TBI. (b, d, f, h) Content of TNF-*α*, IL-1*β*, IL-4, and IL-10 in serum at 24 h after TBI. Data are expressed as the mean ± SEM. *n* = 6. Significance was determined by one-way ANOVA and Dunnett's post hoc analysis. ^∗^*P* < 0.05 versus Sham group; ^#^*P* < 0.05 versus TBI group.

As shown in Figure 5, compared with the Sham group, the levels of TNF-*α* and IL-1*β* in the brain tissue and plasma increased after TBI, while the content of IL-4 and IL-10 significantly reduced (*P* < 0.05, Figures 5(a)–5(h)). After treatment of exogenous irisin, the levels of TNF-*α* and IL-1*β* were significantly lower than those in the TBI group, and the levels of IL-4 and IL-10 significantly increased (*P* < 0.05, Figures 5(a)–5(h)). These results all suggested that exogenous irisin could inhibit the inflammatory response at 24 h after TBI.

**Figure 6:**
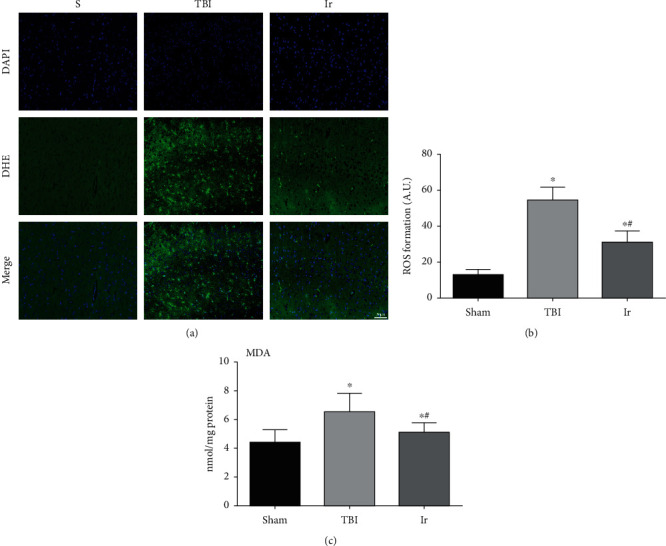
Effects of exogenous irisin on oxidative stress in the brain tissue at 24 hours after TBI in mice. (a, b) The extent of reactive oxygen species (ROS) production in mouse brain tissue was determined by dihydroethidium staining. (c) Measurement of MDA content in mouse brain tissue. Data are expressed as the mean ± SEM. Scale bars: 50 *μ*m. *n* = 6. Significance was determined by one-way ANOVA and Dunnett's post hoc analysis. ^∗^*P* < 0.05 versus Sham group; ^#^*P* < 0.05 versus TBI group.

As shown in Figure 6, compared with the Sham group, the production of ROS in the brain tissue and the MDA content increased significantly after TBI (*P* < 0.05), while with the treatment of exogenous irisin, the ROS production and MDA content were remarkably reduced, compared with the TBI group (*P* < 0.05). These results suggested that exogenous irisin could inhibit oxidative stress after TBI.

**Figure 7:**
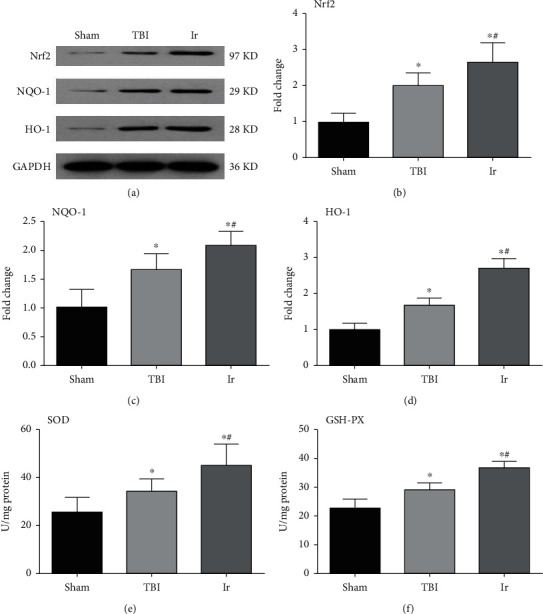
Effects of exogenous irisin on antioxidative response system at 24 hours after TBI in mice. (a–d) The expressions of Nrf2, NQO-1, and HO-1 in brain tissue at 24 h after TBI using western blotting analysis. (e, f) Content of SOD and GSH-PX in brain tissue at 24 h after TBI using ELISA. Data are expressed as the mean ± SEM. *n* = 6. Significance was determined by one-way ANOVA and Dunnett's post hoc analysis. ^∗^*P* < 0.05 versus Sham group; ^#^*P* < 0.05 versus TBI group.

As shown in Figure 7, compared with the Sham group, the expressions of Nrf2 and its downstream proteins NQO-1, HO-1, SOD, and GSH-PX significantly enhanced after TBI (*P* < 0.05, Figures 7(a)–7(f)), suggesting that the oxidative stress was activated. After the treatment of exogenous irisin, the expressions of Nrf2 and its downstream proteins NQO-1, HO-1, SOD, and GSH-PX were significantly higher than those in the TBI group (*P* < 0.05, Figures 7(a)–7(f)), which suggested that exogenous irisin enhanced the intensity of antioxidative stress after TBI.

### 3.6. Exogenous Irisin Reduced Mitochondrial Apoptosis of Vascular Endothelial Cells in the Brain Tissue after TBI

**Figure 8:**
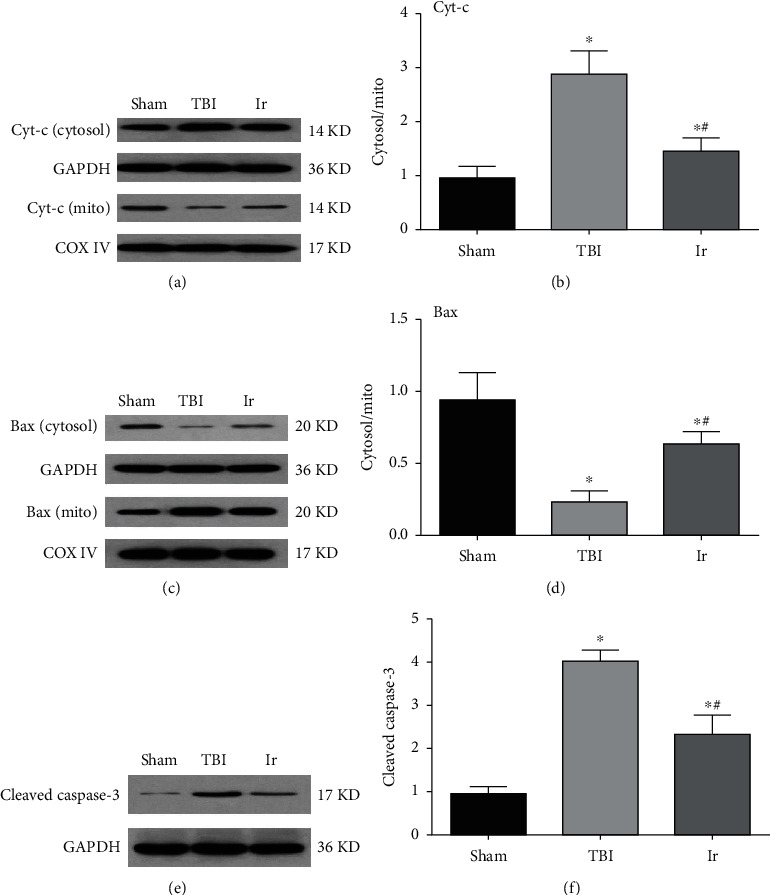
Effects of exogenous irisin on mitochondrial apoptosis at 24 hours after TBI in mice. (a, b) Assessment of Cyt-C in mitochondria and cytosol derived from endothelial cells of mice at 24 hours after TBI. (c, d) Assessment of Bax in mitochondria and cytosol derived from endothelial cells of mice at 24 hours after TBI. (e, f) The expression of cleaved caspase-3 in brain tissue at 24 hours after TBI using western blotting analysis. Data are expressed as the mean ± SEM. *n* = 6. Significance was determined by one-way ANOVA and Dunnett's post hoc analysis. ^∗^*P* < 0.05 versus Sham group; ^#^*P* < 0.05 versus TBI group.

If the typical indicators of endothelial cell mitochondrial apoptosis are detected, it could be further clarified that the effect of exogenous irisin on endothelial cells after TBI and the related mechanism of further influence on BBB permeability could be explored. As shown in Figure 8, compared with the Sham group, Cyt-C of the endothelial cells in the damaged brain region after TBI demonstrated a significantly higher proportion in the cytoplasm and mitochondria (*P* < 0.05, Figures 8(a) and 8(b)). Meanwhile, the ratio of Bax in the cytoplasm and mitochondria significantly reduced while the protein expression of cleaved caspase-3 significantly increased (*P* < 0.05, Figures 8(c)–8(f)). In contrast, compared with the TBI group, the ratio of Cyt-C in the cytoplasm and mitochondria of the Ir group was significantly lower and the ratio of Bax in the cytoplasm and mitochondria was significantly higher, while the protein expression of cleaved caspase-3 significantly dropped (*P* < 0.05, Figures 8(a)–8(f)). The above results suggested that exogenous irisin could slow down the degree to which Bax and Cyt-C transferred after TBI, and lysis of the apoptotic protein caspase-3 was pyrolyzed, thereby reducing the apoptosis of endothelial cells.

### 3.7. Exogenous Irisin Affected Mitochondrial Function by Regulating the Expression of UCP2

**Figure 9:**
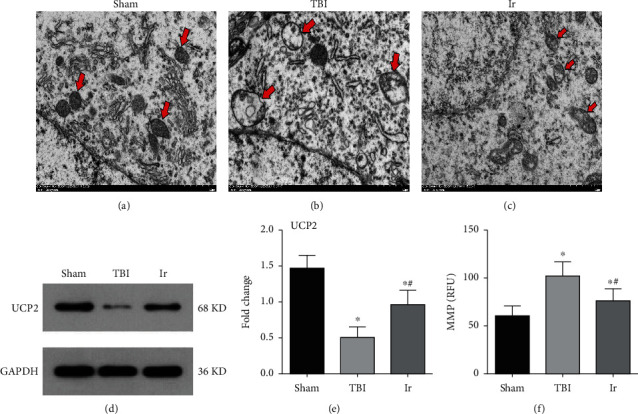
The expressions of UCP2 and ultrastructure of mice at 24 hours after TBI. (a–c) Electron microscopic examination of neuronal mitochondria in mice at 24 hours after TBI. Arrows in red indicate mitochondrial structures of neurons. (d, e) Western blotting analysis of UCP2 in brain tissue at 24 hours after TBI. (f) Assessment of neuronal MMP in mice at 24 hours after TBI. Data are expressed as the mean ± SEM. Scale bars: 1.0 *μ*m. *n* = 6. Significance was determined by one-way ANOVA and Dunnett's post hoc analysis. ^∗^*P* < 0.05 versus Sham group; ^#^*P* < 0.05 versus TBI group.

Uncoupling proteins (UCPs), members of the mitochondrial carrier protein superfamily, are a class of anionic carrier proteins located on the inner mitochondrial membrane. UCP2 located on neurons can regulate the number of mitochondria and mitochondrial membrane potential (MMP) and the absolute value of ATP as well as the generation of oxygen free radicals. Such regulation thereby affects neurotransmission or neurological function and then BBB permeability. As shown in Figures 9(a)–9(c), the mitochondria of neurons in the Sham group had no obvious swelling, uniform size, and normal crista structure. A typical double-layer membrane structure was seen, with a moderate matrix electron density. By contrast, in the TBI group, neuronal mitochondria showed obvious swelling, and their bilayer membrane structures disappeared, inner chamber expanded. Furthermore, the matrix was sparse, and the matrix electron density decreased significantly, showing a vacuole shape, short, and few cristae. Inclusions of different sizes and shapes could be seen in the cristae or matrix. When it came to the Ir group, the degree of neuronal mitochondrial swelling and vacuoles was slightly less than that in the TBI group, and the matrix electron density here tended to be normal. That is, the cristae were visible, and some of the mitochondrial cristae dissolved into small bubbles, with a small number of inclusions seen in the mitochondrial matrix. Compared with the Sham group, the protein expression of UCP2 in the damaged brain area after TBI significantly weakened and MMP significantly increased, while the protein expression of UCP2 upregulated and MMP decreased in the Ir group compared with the TBI group (*P* < 0.05, Figures 9(d)–9(f)).

**Figure 10:**
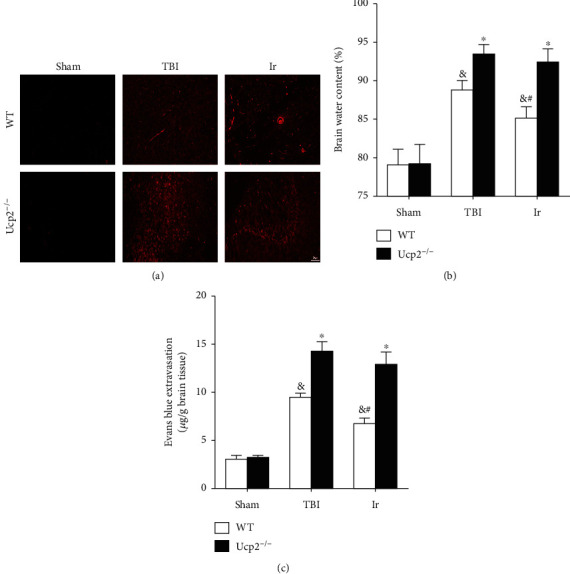
Genetic ablation of UCP2 compromised the protective effect of exogenous irisin on BBB integrity at 24 hours after TBI in mice. (a, c) Evans blue extravasation in WT and UCP2^−/−^ mice at 24 hours after TBI. (b) Evaluation of brain water content in WT and UCP2^−/−^ mice at 24 hours after TBI. Data are expressed as the mean ± SEM. Scale bars: 20 *μ*m. *n* = 6. Significance was determined by one-way ANOVA and Dunnett's post hoc analysis. ^∗^*P* < 0.05 versus WT mice under the same conditions; ^#^*P* < 0.05 versus TBI group in WT mice; ^&^*P* < 0.05 versus Sham group in WT mice.

**Figure 11:**
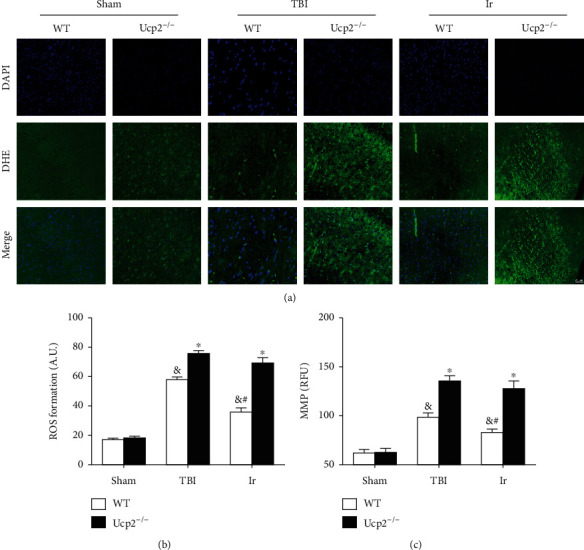
Genetic ablation of UCP2 compromised the protective effect of exogenous irisin on oxidative stress at 24 hours after TBI in mice. (a, b) The extent of reactive oxygen species (ROS) production in WT and UCP2^−/−^ mice was determined by dihydroethidium staining. (c) Assessment of neuronal MMP in WT and UCP2^−/−^ mice at 24 hours after TBI. Data are expressed as the mean ± SEM. Scale bars: 50 *μ*m. *n* = 6. Significance was determined by one-way ANOVA and Dunnett's post hoc analysis. ^∗^*P* < 0.05 versus WT mice under the same conditions; ^#^*P* < 0.05 versus TBI group in WT mice; ^&^*P* < 0.05 versus Sham group in WT mice.

To further testify the correlation between the protective effects of irisin on UCP2, we used UCP2 knockout mice for further experiments. Under normal physiological conditions, there was no difference in BWC, the EB content, ROS level, and neuronal MMP between wild-type mice and UCP2^−/−^ mice (*P* > 0.05, Figures 10 and 11). The increase in BWC, the EB content, and ROS level, which were all caused by TBI, was more obvious in UCP2^−/−^ mice than in wild-type mice (*P* < 0.05, Figures 10 and 11). After treatment with exogenous irisin, the BWC, EB content, ROS level, and neuronal MMP of the UCP2^−/−^ mice were higher than those of the WT mice (*P* < 0.05, Figures 10 and 11).

## 4. Discussion

As the economy and society make advances, recent years have witnessed an increasing incidence of traumatic brain injury (TBI) worldwide, caused by two dominant factors: traffic accidents and falls from heights. There are more than 60 million new cases of TBI patients every year [1, 2]. This is worsened by the fact that the mechanism of the occurrence and development of TBI is complicated, and that, its prognosis is poor, which brings a heavy burden to patients' families and global medical care. Even worse, the specific mechanism of brain edema, cognitive dysfunction, and even Alzheimer's disease caused by TBI is still baffling the global health system [2]. Various animal models have been used to study the pathophysiological changes of TBI to better clinical treatment. This study selected the controlled cortical impact model (controlled cortical impact CCI), which has strong controllability and stability, accurate injury, and controllable impact time to achieve a good simulation of brain edema caused by clinical brain injury [17–21].

Recent studies have proved the remarkable protective effect of physical exercise, particularly aerobic exercise called endurance exercise (EE) on brain injury. EE can effectively alleviate a variety of brain injury including Parkinson's disease, Alzheimer's disease, and neurovascular diseases. This may be attributed to the fact that EE can increase hippocampal blood perfusion, enhance neuronal synaptic plasticity, relieve neuroinflammatory response, and even stimulate neuronal regeneration [10–13]. Although EE's protection on brain injury has been increasingly acknowledged, its specific mechanism remains to be discovered. Studies have found that exercised skeletal muscle secreted irisin, a downstream product of PGC-1*α* protein, and it could make fat browning by promoting the expression of UCP1 [19]. In recent years, it has been reported that irisin exerts a similar protective effect to EE on cerebral ischemia [12, 14]. Consistent with these findings, results in this study showed that the content of irisin in CSF of TBI patients was positively correlated with their prognosis. In addition, mice after exercise or treated with exogenous irisin had better results in their survival rate, cognition, and especially the outcome of cerebral edema after TBI than the TBI group. In our study, we also found that EE could increase the content of irisin in the brain tissue of mice with or without TBI. Thus, all these results indicated that the neuroprotection of EE on TBI may contribute to its promotion of irisin. Meanwhile, it is delightfully noted that the “exercise drugs (irisin)” could effectively replace the beneficial effects of exercise. What is more, we also found that the expression of irisin in the hippocampus was upregulated in the Ir group compared to the TBI group, which indicated that exogenous administration of irisin could promote it to enter the brain tissue. Therefore, the following study was proceeded with exogenous irisin instead of EE to explore the underlying mechanisms.

An important reason for the poor prognosis of TBI is that changes in the permeability of the BBB after TBI lead to cerebral edema [17, 22]. The blood-brain barrier (BBB) refers to a barrier to prevent substances in the blood from entering the brain, formed by brain capillary walls and glial cells. It is located between brain cells and plasma and forms neurovascular units with neurons [21]. BBB is mainly composed of vascular endothelial cells, astrocytes, microglia, pericytes, continuous basement membrane and extracellular matrix, and neurons, among which cerebral microvascular endothelial cells are the main component. Cerebral microvascular endothelial cells are tightly connected with endothelial cells through a unique tight junction (TJ), and they lack openings while having weak endocytosis, so they strictly restrict the paracellular transport, effectively preventing the passage of macromolecules and toxic substances [23].

TJ is mainly composed of two major components: cytoplasmic adhesion protein and transmembrane protein. The former is the basis of TJ's supportive structure, mainly including the ZO series, of which ZO-1 functions most effectively. The main components of transmembrane proteins are occludin and claudin-5, so ZO-1, occludin, and claudin-5 proteins are essential for maintaining the physiological functions of BBB [24, 25]. Recent studies have confirmed that many factors are involved in the mechanism of BBB disruption following TBI. Matrix metalloproteinase (MMP) has a critical role in the pathophysiology of BBB breakdown in TBI, and several investigators have reported the involvement of MMPs in degrading TJ proteins of the BBB [26, 27]. In addition, cytokines, chemokines, and growth factors also play an important role in the pathophysiology of TBI. The inflammatory mediators act through pinocytosis or directly act on the corresponding TJ protein. This causes the structure and function of BBB to be further dysregulated, forming vasogenic brain edema [18, 28]. The results of this study showed that the TJ ultrastructure was destroyed after TBI, the expressions of ZO-1, occludin, and claudin-5 proteins were downregulated, and the inflammatory response was enhanced. With the treatment of irisin, the expressions of ZO-1, occludin, and claudin-5 proteins were upregulated, the degree of TJ ultrastructure damage reduced, and the inflammatory response was inhibited. All these suggested that irisin may alleviate neuroinflammation and enhance the expressions of TJs.

The overwhelming majority of ATP fundamental to life activities are generated in mitochondria by oxidative phosphorylation associated with the coupling of electron transport. However, the oxidative phosphorylation of mitochondria is not completely coupled, and the uncoupling of oxidation and phosphorylation does not produce ATP, which currently attributes to uncoupling proteins on the mitochondria. Uncoupling protein (UCP), a member of the mitochondrial carrier protein superfamily, is a special carrier located on the inner mitochondrial membrane. The UCP superfamily shares a certain similarity in structure and function, and UCP2, UCP4, and UCP5 mainly exist in the nervous system, among which the mechanism of UCP2 in the nervous system has been most fully studied [9, 29]. UCP2 pumps out neuron protons under physiological conditions and lowers the concentration and gradient of protons when back in the mitochondrial matrix, reducing the number of protons flowing through ATP synthase to weaken the synthesis of ATP [30]. Meanwhile, ATP is generated when mitochondrial membrane potential (MMP) sustains oxidative phosphorylation by a potential difference caused by the numerical difference in protons on both sides of the inner membrane [31, 32]. As a result, the activation of UCP2 can reduce MMP. Neuron UCP2 can promote the change of redox state in the cell and reduce the production of mitochondrial ROS and transfer it out of mitochondria by sending redox signals through the interaction with the electron transport chain (ETC). Therefore, neuron UCP2 counts remarkably in reducing the production of ROS and reducing oxidative stress [9], and MMP, to some extent, is positively correlated with the production of mitochondrial ROS [7, 8]. Studies have confirmed that irisin may alleviate mitochondrial damage and reduce the production of ROS to inhibit oxidative stress by regulating the expression of UCP2, thereby reducing lung ischemia-reperfusion injury [33] and severe acute pancreatitis injury [34]. In the present study with the treatment of irisin, the expression of UCP2 was upregulated, the MMP was reduced, and the neuronal mitochondrial damage and oxidative stress alleviated, but these neuroprotective effects were not seen in UCP2^−/−^ mice. Taken together, it was speculated that irisin may alleviate the damage of BBB after TBI and inhibit oxidative stress by promoting mitochondrial UCP2 expression.

Then, how does oxidative stress cause BBB dysfunction after TBI? Under physiological conditions, the production and elimination of active oxygen in the body maintain a constant balance. Excessive ROS production or weakened ability to clean up will lead to the consequence that oxidation in the body is stronger than antioxidation. This results in inflammatory infiltration of neutrophils and increased protease secretion, which generates a large number of active oxygen mediators. Then, oxidative stress damage follows, leading to the death of cells through apoptosis, necrosis, or autophagy [35, 36]. After TBI, impairment is observed in the function of mitochondria in the neurons in the directly damaged region, and neurons release excessive ROS making oxidative stress enhanced [23, 37–39]. These occurrences further lead to opened mitochondrial permeability transition pore (MPTP) of the mitochondrial membrane in endothelial cells of the indirect damaged area, reduced mitochondrial membrane potential (MMP), weakened respiratory chain, swollen mitochondria, and increased membrane permeability. As a result, cytochrome C (Cyt-C) in the mitochondrial membrane is released into the cytoplasm, and the proapoptotic factor Bax transfers from the cytoplasm to the mitochondrial membrane. This causes a cascade reaction of the caspase family, particularly the activation of caspase-3, leading to endothelium apoptosis [40–43]. Capillary endothelial cells in brain tissue constitute the skeleton of BBB, and their apoptosis can lead to the destruction of BBB integrity. In this study, we found that TBI promoted the transfer of Bax in the cytoplasm to the mitochondrial membrane and the release of Cyt-C from the mitochondrial membrane to the cytoplasm, which further led to the lysis of caspase-3 to cause endothelial cell apoptosis, thereby causing BBB dysfunction. In the meantime, irisin slowed down the degree of Bax and Cyt-C metastasis after TBI reduced the expression of caspase-3 and reduced the degree of endothelial cell apoptosis. Taking these into consideration, we speculated that irisin could reduce mitochondrial-mediated ROS production and inhibit oxidative stress by promoting UCP2 expression.

Admittedly, this study still needs further perfection in the following aspects. First of all, the number of research participants (TBI patients) in our clinical research is limited, and the period for observing their prognosis after TBI is not long enough, restricted by objective condition. This may be a stimulus for future studies to establish a multicenter study for a larger sample size. Furthermore, this study fails to testify whether there is any significant difference in irisin content and prognosis between the EE group and the nonexercise group after TBI. This appeals to further studies since exercises can stimulate the release of irisin in the body. Moreover, more attention is needed to address the issue that we focused on the damage in function and structure of mitochondria in neurons and vascular endothelial cells after TBI, but actually, the mitochondria of other cells in the brain tissue were also damaged to varying degrees. This will also be covered in our further studies. Finally, it remains to be elucidated in future research whether UCP4 or UCP5 was also associated with the destruction of structure and function of BBB after TBI.

**Figure 12:**
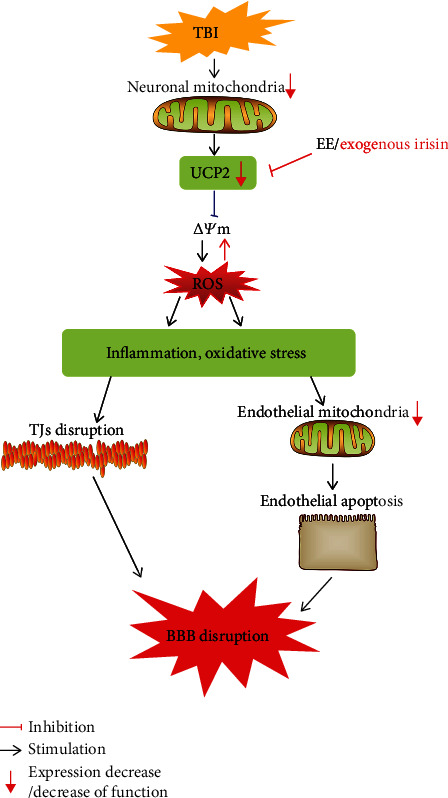
EE/exogenous irisin alleviates BBB disruption after TBI via the suppression of oxidative stress and neuroinflammation.

In summary, this study has concluded that EE may reduce the BBB damage caused by TBI through promoting the secretion of irisin in brain tissue, and its effect was similar to the treatment of exogenous irisin. In addition, we also found that irisin could exert its neuroprotection by promoting the expression of UCP2 on the mitochondrial membrane to improve the dysfunction of BBB after TBI by ultimately alleviating inflammation and oxidative stress (Figure 12).

## Data Availability

All data presented in the present study are available from the corresponding author on reasonable request.
